# l-Peptide functionalized dual-responsive nanoparticles for controlled paclitaxel release and enhanced apoptosis in breast cancer cells

**DOI:** 10.1080/10717544.2018.1477863

**Published:** 2018-05-30

**Authors:** Shiwei Niu, David H. Bremner, Junzi Wu, Jianrong Wu, Haijun Wang, Heyu Li, Qianqian Qian, Hong Zheng, Limin Zhu

**Affiliations:** a College of Chemistry, Chemical Engineering and Biotechnology, Donghua University, Shanghai, PR China;; b School of Science, Engineering and Technology, Abertay University, Dundee, Scotland, UK;; c Department of Experimental Animal Science, Kunming Medical University, Kunming, PR China

**Keywords:** l-Peptide, dual-responsive, apoptosis, nanoparticles, anticancer efficacy

## Abstract

Nanoparticles and macromolecular carriers have been widely used to increase the efficacy of chemotherapeutics, largely through passive accumulation provided by their enhanced permeability and retention effect. However, the therapeutic efficacy of nanoscale anticancer drug delivery systems is severely truncated by their low tumor-targetability and inefficient drug release at the target site. Here, the design and development of novel l-peptide functionalized dual-responsive nanoparticles (l-CS-g-PNIPAM-PTX) for active targeting and effective treatment of GRP78-overexpressing human breast cancer *in vitro* and *in vivo* are reported. l-CS-g-PNIPAM-PTX NPs have a relative high drug loading (13.5%) and excellent encapsulation efficiency (74.3%) and an average diameter of 275 nm. The release of PTX is slow at pH 7.4 and 25 °C but greatly accelerated at pH 5.0 and 37 °C. MTT assays and confocal experiments showed that the l-CS-g-PNIPAM-PTX NPs possessed high targetability and antitumor activity toward GRP78 overexpressing MDA-MB-231 human breast cancer cells. As expected, l-CS-g-PNIPAM-PTX NPs could effectively treat mice bearing MDA-MB-231 human breast tumor xenografts with little side effects, resulting in complete inhibition of tumor growth and a high survival rate over an experimental period of 60 days. These results indicate that l-peptide-functionalized acid – and thermally activated – PTX prodrug NPs have a great potential for targeted chemotherapy in breast cancer.

## Introduction

Breast tumors, characterized by unlimited proliferation, is a life-threatening disease with high morbidity and mortality (Fan et al., 2017; Tiash et al., 2017). Conventionally, the disease is clinically treated through chemotherapy and one of the most successful compounds for the treatment of extensive tumors (e.g. breast, lung, liver, kidney, etc.) (Tiwari et al., 2017) is the diterpenoid, paclitaxel (PTX), mainly extracted from the bark of the Pacific yew tree (Jiang et al., 2016). PTX has demonstrated outstanding antitumor activity due to high-affinity binding to microtubules, stabilizing and enhancing tubulin polymerization, and as suppressors of spindle microtubule dynamics (Wang et al., 2016; Zhang et al., 2018). Subsequently, all those actions contribute to the suppression of cell mitosis, motility and intracellular transport, which lead ultimately to the apoptosis of carcinomas (Lin et al., 2017). Due to the above properties, PTX has frequently been employed as a model drug among a number of important chemotherapeutic agents in recent cancer research (Yuan et al., 2015). However, most antitumor drugs exhibit high-dose-limiting toxicities and narrow therapeutic windows because of their poor specificity and inadequate targeting ability (Zhang et al., 2017). Likewise, in clinical trials, PTX causes a series of side effects, such as hypersensitivity, myelosuppression, and neurotoxicity, and many advances using PTX have been limited by its poor aqueous solubility (Kasala et al., 2017). Furthermore, the most detrimental aspect is that intravenous administration of PTX results in drugs accumulating, not only in the lesion site but also in other normal tissues, therefore, the selection of appropriate carriers for drug delivery is particularly critical for the clinical application of this antitumor compound (Pi et al., 2017). In order to resolve these difficulties, nano-formulations have been the focus of much research in order to improve the solubility and targeting efficiency of PTX in the field of anti-tumor drug therapeutics.

Among various PTX delivery systems, nano-scale biopolymers hold numerous advantages (Bhirde et al., 2010; Madane & Mahajan, 2016; Zhong et al., 2016; Huang et al., 2017), including their ability to improve drug stability and pharmacological properties, offer enhanced longevity in the blood and they provide an eligible carrier system that can protect and effectively deliver drugs to the target site by integrating different treatment strategies into one platform in order to minimize side effects. Specifically, compared with conventional medicines, nanoparticle drug delivery systems are able to exhibit passive/active target properties and controlled or sustained drug-release kinetics (Xie et al., 2016). These nanoparticles can also enhance the physicochemical properties of drugs due to their enhanced permeability and retention (EPR) effect and low cytotoxicity (Chen et al., 2016) as well as promoting gradual apoptosis of distinctive carcinomas. For passive targets, it is notable that the observed tumor environments are characterized by mild hyperthermia (1–2 °C above healthy tissue), acidic pH (lower than usual cellular pH) (Canning et al., 2017) and excessive cell proliferation accompanied by high enzymatic concentration compared to normal tissues, which neutralizes the therapeutic efficacy of administering the cytotoxic drug. Consequently, over the past 25 years, a great deal of research has concentrated on stimuli-responsive polymers in order to obtain materials able to respond to specific surroundings (Duan et al., 2011). These drug delivery carriers are temperature- or pH-responsive, and are frequently called ‘smart polymers’ (Zhang & Misra, 2007) as they are able to undergo fast, abrupt, and reversible alteration in their structure/properties in a response to small changes in their surroundings. In order to improve and increase their applicability, several smart polymers have been developed to combine two or more mechanisms of responsiveness in only one platform. In the present study, the combination of pH – and temperature – responsive properties were generated by means of graft copolymerization of chitosan (CS) with poly(*N*-isopropylacrylamide) (PNIPAM) so as to carry out these dual-responsive functions concurrently. As is well known (Chan et al., 2007), CS (2-amino-2-deoxy-(1 → 4)-β-d-glucosamine), which is obtained from chitin by deacetylation, is considered to be one of the most widely distributed cationic biopolymers (Ding et al., 2017). It has good biocompatibility and biodegradability and has been used extensively in the preparation of safe biomaterials. Chitosan nanoparticles (CS NPs) offer numerous advantages making them promising drug delivery materials (Hua et al., 2011). Due to their subcellular and sub-micrometer size, CS NPs can penetrate deeply into tissues, and pass through the fenestration present in the epithelial lining (Hwang et al., 2008) and, above all, CS is sensitive to variations in pH (Hashad et al., 2016). These factors indicate that CS a perfect material for delivery of therapeutic agents into the body. On the other hand, PNIPAM is widely recognized as a thermal responsive polymer, since the lower critical solution temperature (LCST) of PNIPAM is closer to body temperature (Echeverria et al., 2015) and it undergoes a volume transition in water upon heating above 32 °C. Additionally, PNIPAM is biodegradable, therefore, PNIPAM modified CS has been synthesized as the dual-responsive polymer for promising applications in the biomedical field. In this paper, chitosan-graft-poly(*N*-isopropylacrylamide) (CS-g-PNIPAM) NP was employed as a potential antitumor drug carrier that could respond to both temperature and pH simultaneously. In the past few decades, many grafting modifications for the preparation of thermally sensitive CS materials, such as living free radical nitroxide-mediated polymerization (NMP) and atom transfer radical polymerization (ATRP) have been reported, however, these polymerizations were not controlled. In contrast, reversible addition fragmentation chain transfer (RAFT) polymerization, which is a fast reversible process involving a thiocarbonyl compound reacting with propagating chain radicals, may be a promising alternative (Tang et al., 2008). In this study, the RAFT technique was employed to synthesize CS-g-PNIPAM NPs.

**Scheme 1.**
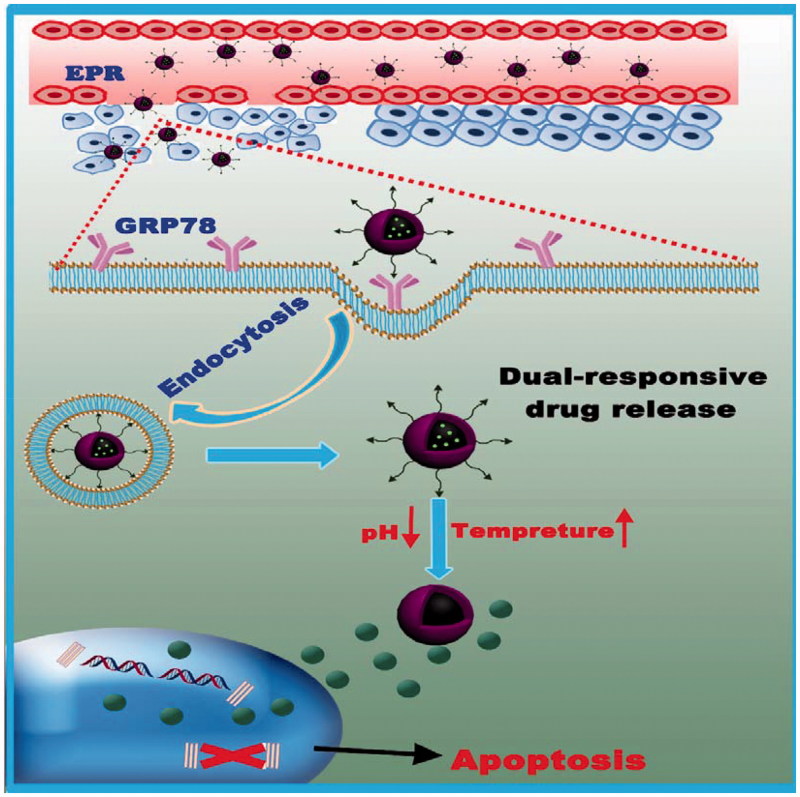
Schematic illustration of the smart NPs with prolonged blood circulation, enhanced tumor accumulation, efficient cancer cell uptake, pH- and temperature-responsive release of PTX, and the capability of targeting breast cancer cells.

However, to act as versatile platforms for cancer therapy, the specificity of the nanoparticles toward tumors requires further improvement. An optimal nanoparticle system should have the ability to ‘turn off’ the internalization function during circulation in the blood, but be ‘turned on’ when inside a tumor. Recently (Chen et al., 2016; Wang et al., 2016), active strategies using biological targeting moieties, such as peptides, proteins, monoclonal antibodies, nucleic acid aptamers, and other small molecules (Gao et al., 2009; Hu et al., 2011) which bind to specific biomarkers on the surface of tumor cells have been developed. In principle, these active targeting strategies involve modifying the surface of nanoparticles with target molecules that can recognize and bind specifically to the tumor cells (Goel et al., 2014; Shamay et al., 2016), enhancing the ability of the nanoparticles to be transported into the tumor cells from the extracellular space (Gao et al., 2009, 2016; Goel et al., 2014). Peptide ligands provide a promising prospect as an active target probe and are commonly discovered by selection from a random peptide library using display technologies or from the sequences of the binding proteins (He et al., 2017). Here a tumor-targeting peptide was proposed previously (Lee et al., 2004), l-peptide (RLLDTNRPLLPY), which was discovered through screening phage-display peptide libraries on nasopharyngeal carcinoma (NPC) cells is utilized. In addition, it is reported that l-peptides might bind GRP78 (glucose-regulated protein 78, a verified specific tumor surface marker as well as a promising target for selective cytotoxicity of carcinomas) on the surface of cancer cells (Wang et al., 2016) and l-peptide-linked liposomes loaded with doxorubicin showed a higher efficacy for tumor suppression than liposomal doxorubicin alone, and apparently did not cause side effects *in vivo* (Wang et al., 2016). Also, further investigation is required since GRP78 plays an anti-apoptotic role in excessive proliferative cells (Chen et al., 2014), therefore, l-peptide decorated NPs may regulate both pro-survival and pro-apoptotic signalingpathways in tumors. In the present study, the l-peptide was conjugated to the CS-PNIPAM NPs as a drug carrier, endowing it with passive and active targeting properties simultaneously, and after loading this formulation with PTX, employing it in breast cancer therapy (Scheme 1). Subsequently, the stimuli-responsive behavior of this bio-copolymer, the capacity of this peptide-functionalized NP to target cancer cells, as well as the therapeutic efficacy of this versatile formulation was examined. Moreover, on the basis of the experimental results, considerable effort has been made to achieve an optimal tumor-targeting effect, and to offer the possibility of a viable breast tumor chemotherapy regime to the clinician.

## Materials and methods

### Materials

CS (degree of deacetylation >90%, Mw ∼ 200 kDa) was obtained from Sino Pharm Chemical Reagent Co., Ltd (Shanghai, China). Azobisisobutyronitrile (AIBN), *S*-1-dodecyl-*S*′-(α, α′-dimethyl-α″-acetic acid) trithiocarbonate (DDACT) and PTX were purchased from the Aladdin Company (Shanghai, China). Hoechst 33342 was obtained from the Keygen Biochemical Company (Nanjing, China). *N*-Isopropylacrylamide (NIPAM), fluorescein isothiocynate (FITC), N, N-dicyclohexylcarbodimide (DCC), 4-(dimethyl-amino) pyridine (DMAP), 1-(3-dimethylaminopropyl)-3-ethylcarbodiimide hydrochloride (EDC.HCl), *N*-hydroxysuccinamide (NHS), 3-(4, 5-dimethyl-thiazol-yl)-2, 5-diphenyltetrazolium bromide (MTT), and phosphate buffered saline (PBS) were purchased from Sigma-Aldrich (St. Louis, MO). N, N-Dimethyl formamide (DMF) was distilled under reduced pressure from calcium hydride and stored over molecular sieves (4A). l-Peptide (RLLDTNRPLLPY) was provided by Dangang Bio-Technology Co., Ltd. (Hangzhou, China). Dulbecco's Modified Eagle Medium (DMEM), fetal bovine serum (FBS), penicillin, streptomycin, and trypsin–EDTA were obtained from Gibco (Carlsbad, CA). MDA-MB-231 cells and L929 cells were purchased from the Type Culture Collection of the Chinese Academy of Sciences (Shanghai, China). Deionized water used throughout the research work was produced using a Milli-Q Gradient A10 System. All other chemical reagents were of analytical grade and used without further purification.

Female nude mice (aged 4–6 weeks) were provided by the Animal Center of Kunming Medical University (Kunming, China). The animals were housed at 20–22 °C with a 12:12 h light/dark cycle. The protocol of the experiment was approved by the Animal Care and Use Committee of Kunming Medical University. All animal experimental procedures conform to the Guide for the Care and Use of Laboratory Animals that was published by the US National Institute of Health (NIH Publication No. 8523, revised 1985).

### Synthesis of l-peptide functionalized CS-g-PNIPAM NPs

**Figure 1.**
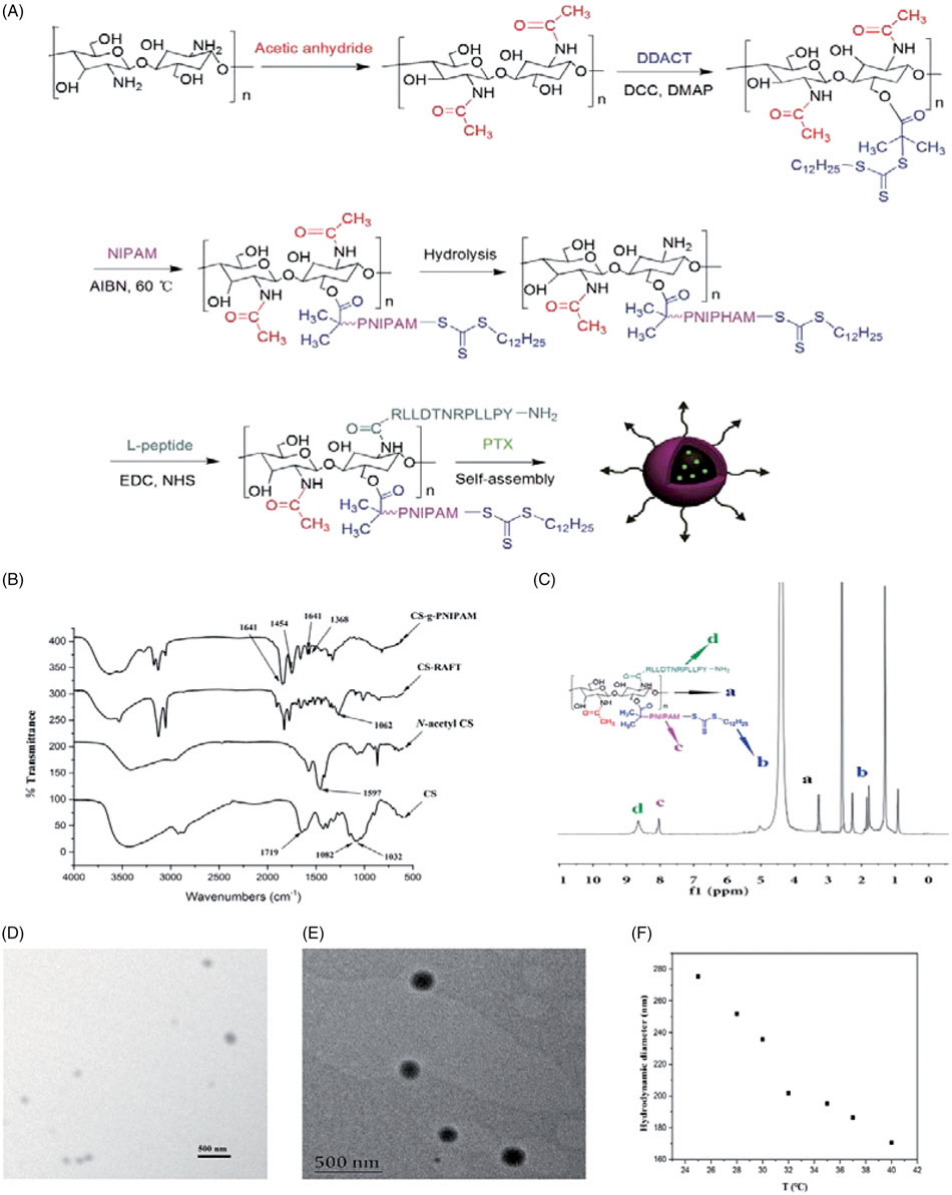
(A) Schematic representation of synthesis of l-CS-g-PNIPAM-PTX NPs. (B) FT-IR spectra of CS, *N*-acetyl CS, CS-RAFT, and CS-g-PNIPAM. (C) ^1^H NMR spectrum of l-CS-g-PNIPAM NPs. (D) Representative SEM micrograph of l-CS-g-PNIPAM-PTX NPs; and (E) representative TEM micrograph of l-CS-g-PNIPAM-PTX NPs; (F) evolution of the measured mean hydrodynamic diameters of l-CS-g-PNIPAM-PTX NPs using the tested range of temperatures.

The preparation of the PTX-loaded l-peptide functionalized CS-g-PNIPAM (l-CS-g-PNIPAM-PTX) NPs was based on the RAFT method (Tang et al., 2008), using the synthetic route shown in Figure 1. Initially, in order to protect the NH_2_– functionality, CS (5 g) was dissolved in acetic acid (500 mL; 1.0%) containing acetic anhydride in the molecular ratio (1:0.4). After the addition of absolute ethanol (500 mL), the mixture was stirred at room temperature under nitrogen and after 8 h of reaction, the resulting mixture was poured into ice water. The precipitate was then filtered, washed with of methanol (150 mL) and dried at room temperature for 1 h to obtain *N*-acetyl CS. Subsequently, the CS-RAFT agent was prepared according to the previously described procedure (Tang et al., 2008). *N*-acetyl CS (0.29 g) was dissolved in dry DMF (30 mL) and stirred for 48 h at room temperature with DDACT (1.0 mmol) in the presence of DCC (1.0 mmol) and DMAP (0.12 mmol). The resulting mixture was poured onto ice water, the precipitate was filtered, then washed completely by Soxhlet extraction with acetone and dried to give a yellow powder (0.499 g, 81% yield). A mixture of the CS-RAFT agent (0.05 mmol trithio groups) and dry DMF (5 mL) was stirred magnetically under argon atmosphere until completely dissolved. AIBN (0.0016 g, 0.01 mmol) and NIPAM (0.50 g) were added, the reaction mixture was heated to 60 °C with stirring for 24 h and then the reaction mixture was poured into diethyl ether (25 mL) and the precipitate filtered to obtain *N*-acetyl CS-PNIPAM NPs (0.0295 g, 63% yield). The acetyl groups were removed by stirring the *N*-acetyl CS-PNIPAM NPs in NaOH solution (15.4 M) 48 h at room temperature. Covalent binding of the l-peptide (NH_2_-RLLDTNRPLLPY-COOH) was achieved by first protecting the NH_2_ by acetylation and then linking this to the NH_2_-CS-PNIPAM by activating with EDC/NHS (EDC 104 mg, NHS 68 mg) solution for 30 min and then adding the l-peptide (14.28 mg/mL, 1.5 μL) to form a mixed solution which was reacted under gentle stirring at room temperature overnight. After hydrolysis of the N-acetyl group, the resulting mixture was centrifuged at 11,000 rpm for 10 min and washed three times with DI water in order to remove any unreacted starting materials. Finally, PTX-loaded NPs were prepared by the solvent dialysis method whereby the freeze-dried l-CS-g-PNIPAM NPs (100 mg) and PTX (20 mg) were dissolved in DMSO (10 mL), sealed in a dialysis bag with a 14,000 Da Mw cutoff, dialyzed for 48 h at 25 °C to remove any small molecules and the resultant solution was centrifuged at 6000 rpm for 30 min to remove any un-encapsulated drug.

### Drug loading and entrapment efficiency

As previously reported (Li et al., 2009, 2017), the amount of encapsulated PTX was determined using the HPLC solvent composition method. Briefly, l-CS-g-PNIPAM-PTX NPs (15 mg) were dissolved in the HPLC eluent and centrifuged at 6000 rpm for 15 min; filtered through 0.22 μm membrane filter; and the supernatant was analyzed for PTX by HPLC. The percentage encapsulation efficiency (EE) and drug loading (DL) were calculated as follows:

$$\begin{array}{l} \%\text{EE}=({\text{PTX}}_{\text{t}}-{\text{PTX}}_{\text{f}}/{\text{PTX}}_{\text{t}})\times 100\\ \%\text{DL}=({\text{PTX}}_{\text{t}}-{\text{PTX}}_{\text{f}}/\text{Total weight of nanoparticles})\times 100 \end{array}$$

where PTX_t_ is the total amount of PTX used in the preparation of NPs and PTX_f_ is the un-encapsulated PTX present in the supernatant.

### Characterization of NPs

Fourier transform infrared (FT-IR) spectra were recorded on a Nicolet Nexus 670 spectrometer (Thermo Fisher, Waltham, MA) by using powdered NPs (1 mg) dispersed in KBr (100 mg). Scans were recorded over 3500–1125 cm^−1^ with the resolution of 2 cm^−1^ and an average of 32 scans. ^1^H spectral measurements were performed on a Bruker DRX 400 nuclear magnetic resonance spectrometer (Bruker, Fällanden, Switzerland) using D_2_O or d_6_-DMSO as solvent. The mean hydrodynamic particle size and the polydispersity index (PDI) were measured by dynamic light scattering (DLS, 90 Plus Particle Size Analyzer, Brookhaven Instruments, Holtsville, NY). Zeta potential (ZP) was measured by the same instrument equipped with a ZP analyzer as previously described (Song et al., 2017). The particle size, PDI, and ZP were measured in triplicate. Particle morphology was characterized with a scanning electron microscope (SEM) (FEI Nova TM Nano SEM, FEI, Hillsboro, OR) and transmission electron microscopy (TEM) (JEOL 2010F, Tokyo, Japan) operating at 200 kV using an aqueous solution of the sample (3 mg/mL) dropped onto a carbon-coated copper grid and air dried before TEM analysis.

### *In vitro* drug release

*In vitro* release behaviors of PTX, as a model drug, from the NPs were studied by a dialysis method (Yang et al., 2012). When the pH-responsive property was studied, the lyophilized PTX-loaded NPs (containing 1 mg PTX) were added to PBS (1 mL; pH =7.4) or acetate buffer (1 mL; pH = 5.0) in a dialysis bag (molecular weight cutoff: 8000–14,000 Da), which was then immersed in the same buffer medium (25 mL) and magnetically stirred (100 rpm) at 37 °C. At predetermined times, aliquots (1 mL) were taken from the medium and replaced with pre-heated buffer solution (1 mL) to maintain a constant volume and the amount of PTX released was determined by HPLC analysis as previously described. Likewise, in an attempt to demonstrate the temperature-responsive structure changes facilitating PTX release of this NP, analogous experiments were performed in PBS (pH 7.4; 0.1 M) at 25 °C and 37 °C. Each experiment was repeated in triplicate.

### Cell cultures

Breast cancer cell line MDA-MB-231 and fibroblast cell line L929 used in this study were cultured in 25 mL flasks and maintained in a humidified 5% CO_2_ incubator at 37 °C, with DMEM containing 100 U/mL penicillin and 100 μg/mL streptomycin and 10% FBS. All cells were sub-cultivated approximately every three days at 80% confluence using 0.25% (w/v) trypsin at a split ratio of 1:5.

### Western blot assay

MDA-MB-231 and L929 cells were washed and lysed in modified RIPA buffer supplemented with 1:100 (v/v) of the proteinase/phosphatase inhibitor cocktail (Solarbio, Beijing, China). Insoluble material was removed by centrifugation at 12,000×*g* at 4 °C for 30 min. Protein was determined by a BCA commercial kit (Sigma, St. Louis, MO) and an equal amount of total protein (40 µg) was loaded per lane and separated on a 10% SDS-PAGE. Proteins were then transferred to polyvinylidene difluoride (PVDF) membranes. Primary antibodies were anti-GRP78 (1:2000 dilution) and anti-β-actin (1:5000 dilution) antibodies (Santa Cruz Biotech, Santa Cruz, CA). The secondary antibody was a horseradish peroxidase (HRP)-conjugated anti-rabbit or mouse IgG (1:5000 dilution; Santa Cruz Biotech, Santa Cruz, CA). The membranes were detected by enhanced chemiluminescence (Millipore, Burlington, MA) and exposed to an X-Omat film (Kodak, Xiamen, China), developed and the intensity of the immunoreactivity was measured by densitometry using Image J software.

### *In vitro* cytotoxicity assays

The cytotoxicity of the PTX-loaded NPs against the above-mentioned MDA-MB-231 cell lines was assessed by using an MTT assay, using L929 cell lines as control. MDA-MB-231 and L929 cells were continuously grown in DMEM cell culture medium supplemented with penicillin (100 U/mL) and streptomycin (100 µg/mL). Subsequently, confluent cells were collected and seeded in 96 well plates at a density of ∼1 × 10^4^ cells per well. After 24 h, prescribed amounts of NPs (i.e. Taxol, l-CS-g-PNIPAM, and l-CS-g-PNIPAM-PTX) at PTX concentrations ranging from 0.001 to 10 µg/mL in 20 µL of PBS were added and incubated in an atmosphere containing 5% CO_2_ for 4 h at 37 °C, then the supernatant was carefully aspirated, and the MTT-formazan generated by live cells was dissolved in DMSO (150 µL) over 20 min. Finally, the absorbance at 490 nm was measured using a microplate reader (MULTSIKANMK3, Thermo, Waltham, MA). The cell viability (%) was determined by comparing the absorbance at 490 nm with control wells containing only cell culture medium. The experiments were performed in triplicate.

### *In vitro* cellular uptake

To verify the targeting capacity of l-peptide, cellular uptake experiments were employed. The NPs were previously labeled with FITC as follows: the CS-g-PNIPAM-PTX or l-CS-g-PNIPAM-PTX NP (100 mg) was dissolved in CH_3_OH, then the activated FITC (40 mg) was added to the mixture, which was stirred at room temperature in the dark for 12 h. The resultant mixture was purified by recrystallization from acetone. The purified CS-g-PNIPAM-PTX/FITC and l-CS-g-PNIPAM-PTX/FITC were obtained as faint yellow powders in 68% and 75% yield, respectively. The cell internalization study of l-CS-g-PNIPAM NPs utilized L929 and MDA-MB-231 cells seeded at a density of ∼1.0 × 10^4^ cells per well on 18 mm coverslips. After 24 h of incubation, the old medium was removed and fresh medium (2 mL) containing 200 μL solution of CS-g-PNIPAM-PTX NPs or l-CS-g-PNIPAM-PTX NPs (1 mg/mL) was added. The medium was discarded after 3 h, the cells were washed with PBS (pH 7.4) for 15 min and the washing step was repeated twice. Then, the cells were fixed with chilled methanol (–20 °C) and stained with Hoechst 33342 (1 µg/mL); the cells, were washed twice with PBS to remove excess dye; and the coverslips were mounted on glass slides with glycerol. Finally, the fluorescent images of the cells were observed using fluorescence microscopy (Nikon Eclipse Ti-S, Nikon Ltd, Tokyo, Japan).

The flow cytometry assay was carried out to verify the specific cellular uptake capacity of l-CS-g-PNIPAM-PTX NPs. L929 and MDA-MB-231 cells were inoculated into a six-well cell culture plate and co-incubated with 200 μL CS-g-PNIPAM-PTX NPs or l-CS-g-PNIPAM-PTX NPs (1 mg/mL) for 4 h, respectively. Meanwhile, the group co-incubated with PBS was used as control. Subsequently, all the cells in each plate were washed twice with PBS, collected and the fluorescence intensity of PTX in each sample was measured by flow cytometry.

### Molecular level evaluation of apoptosis

Molecular level evaluation of apoptosis was performed by a real-time reverse transcriptase polymerase chain reaction (RT-qPCR). Three representative pro-apoptotic genes (*Bax*, Caspase-3, *Caspase-8*) and one anti-apoptotic gene (*Bcl-2*) were selected for determination at the messenger RNA (mRNA) level. MDA-MB-231 cells (1 × 10^6^ cells/mL) were harvested after being incubated with PBS, PTX, and l-CS-g-PNIPAM-PTX for 48 h. The intracellular total RNA was extracted using RNeasy Plus Mini Kits (Qiagen, Hilden, Germany) according to the manufacturer’s instructions and random primed cDNAs were generated by reverse transcription of total RNA samples with SuperScript II reverse transcriptase (Thermo Scientific, Waltham, MA). Quantitative real-time PCR was performed using the ABI Prism 7300 Sequence Detection System (Applied Biosystems, Foster City, CA). The PCR mix contained cDNA (2 µL), of the appropriate forward and reverse primers (1 µL) and SYBR Green PCR Master mix (10 µL) (Roche, Basel, Switzerland) in a total volume of 20 µL. PCR consisted of 40 cycles of denaturation at 94 °C for 30 s, annealing at melting temperature (Tm) for 30 s and extension at 72 °C for 60 s. Primer sequences were designed as follows: *Bax* forward primer 5′-TCATGGGCTGGACATTGGAC-3′ and reverse 5′-GAGACAGGGACATCAGTCGC-3′, *Caspase-3* forward primer 5′-CGGCGCTCTGGTTTTCGTTA-3′ and reverse 5′-CAGAGTCCATTGATTCGCTTCC-3′, *Caspase-8* forward primer 5′-CTGGTCTGAAGGCTGGTTGT-3′ and reverse 5′-GTGACCAACTCAAGGGCTCA-3′, *Bcl-2* forward primer 5′-CAACATCGCCCTGTGGATGA-3′ and reverse 5′-GGGCCAAACTGAGCAGAGTC-3′, and *β-actin* forward primer 5′-CGGCGCCCTATAAAACCCA-3′, and reverse 5′-CGCGGCGATATCATCATCCA-3′ (Tm 60 °C). The fluorescence signal was harvested at the end of each cycle and the results were analyzed by the 2^–△△CT^ method.

### Hemolysis study

An *in vitro* blood hemolysis study was performed in order to determine the pharmacological safety of the l-CS-g-PNIPAM-PTX NPs. Whole blood was harvested from Sprague–Dawley rats and centrifuged at 1500 rpm for 15 min to isolate the red blood cells (RBCs), which were then washed three times with cold saline (Zhang et al., 2016). Taxol (PTX in Cremophor EL and ethanol, v/v 1:1), l-CS-PNIPAM and l-CS-g-PNIPAM-PTX were serially diluted to different concentrations and incubated for 30 min or 120 min at 37 °C in a water bath. After incubation, intact RBCs were separated by centrifugation (1500 rpm for 10 min) and supernatant was collected. The amount of hemoglobin release was determined by reading the absorbance at 540 nm in a microplate reader (MULTSIKANMK3, Thermo, Waltham, MA). To determine 100% hemolysis, RBCs were lysed with ultrapure water and the hemolysis ratio (HR, %) was calculated relative to ultrapure water, whereas the saline acted as a negative control. Lower than 10% of HR was recognized as being biocompatible in this study.

### *In vivo* antitumor efficacy of l-CS-PNIPAM-PTX NPs

The antitumor efficacy of the l-CS-PNIPAM-PTXNPs was evaluated in tumor-bearing xenografts. A total of thirty 6-week-old female athymic nude mice were used in this experiment and each mouse was subcutaneously injected with a suspension of 5 × 10^6^ MDA-MB-231 human breast tumor cells in physiological saline (100 µL). When the tumor volume reached ∼100 mm^3^, mice were divided into three groups (*n* = 10) randomly, and subjected to one of the following treatments: (a) physiological saline, (b) free PTX (dissolved in DMSO), (c) l-CS-PNIPAM-PTX (10 mg PTX equiv./kg) via a tail vein every seven days. The body weight and survival rate were recorded, tumor growth was monitored for each tumor-bearing mouse every other day. At the end of the experiments, the animals were sacrificed, the tumors were removed, and subjected to metrological analysis. The tumor volume was calculated by the following formula: *V* = (*L* × *W* ^2^) × 0.5, where *L* is the length and *W* is the width of the tumor.

### TUNEL and histological examination

To detect cell apoptosis and morphological changes in tumor tissue, tumor tissues isolated from the xenografts were sliced into thin sections and stained with a TUNEL apoptosis detection kit (EMD Chemicals Inc., Darmstadt, Germany). For histological examination, the samples were fixed with 4% formalin, embedded with paraffin and sliced into thin sections which were stained with hematoxylin and eosin (H&E) and observed by an optical microscope.

### Statistical analysis

All experiments were repeated at least three times. Data are expressed as the mean ± standard deviation. Data were analyzed for statistical significance using ANOVA followed by Tukey’s post hoc test (SPSS 19.0; SPSS, Chicago, IL). *p* Values less than .05 were considered to be statistically significant.

## Results

### Synthetic route

As a prerequisite condition, required constitutional units are essential for the reliable fabrication of smart multifunctional nanocarriers through controlled self-assembly of dual-responsive block copolymers (Chen et al., 2016). In the present study, the preparation of two kinds of functional monomers, including pH-responsive CS and temperature-responsive NIPAM was the initial starting point and the synthetic route is briefly illustrated in Figure 1(A). The dual-responsive NPs of CS-g-PNIPAM were obtained from the monomers of NIPAM and CS by RAFT copolymerization in the presence of DDACT as a cross-linking agent.

### The preparation and characteristics of various kinds of NPs

First, in order to prepare the CS-RAFT agent, the CS was successively modified with acetic anhydride to protect –NH_2_ groups of the CS and, subsequently, *N*-acetyl CS was reacted with DDACT in the presence of DCC and DMAP to afford the CS-RAFT agent. NIPAM was then grafted onto the CS-RAFT according to the previously described method (Hua et al., 2008) and FT-IR and ^1^H NMR techniques were applied to confirm the identity of the synthesized copolymers. The FT-IR spectra of original CS, the *N*-acetyl CS, the CS-RAFT agent and the grafted CS-g-PNIPAM copolymers are shown in Figure 1(B). The absorption bands at 1082 and 1032 cm^−1^ can be ascribed to the C3–OH and C6–OH of the CS and it can be clearly seen that the characteristic peaks of CS at 1597 cm^−1^ (–NH_2_) have disappeared after the successful synthesis of CS-*g*-PNIPAM. In comparison with the original CS, the peak at 1719 cm^−1^ (C-O) appeared for chitosan-RAFT agent, while the absorption for the trithiocarbonate units (1062 cm^−1^) was not observed due to the overlap with the strong absorption of CS between 950 cm^−1^ and 1200 cm^−1^. For CS-*g*-PNIPAM, the characteristic peaks at 1454 cm^−1^ (–CH_3_) and 1641 cm^−1^ (C-O) showed the presence of PNIPAM, and their intensity was greatly enhanced in the case of CS-*g*-PNIPAM. In addition, the copolymers showed new absorptions at 1385 and 1368 cm^−1^ compared with CS, suggesting the presence of isopropyl groups in NIPAM. All these results further demonstrated the successful synthesis of CS-*g*-PNIPAM.

Furthermore, the successful introduction of NIPAM and the l-peptide onto CS was verified by ^1^H NMR and the spectrum of l-CS-g-PNIPAM NPs (Figure 1(C)) displays the peaks of the protons on the carbon of CS (at 3.00–4.00 ppm). It is evident that the characteristic resonances of PNIPAM are shown at 0.82–2.10 ppm and 7.80 ppm, suggesting that CS-*g*-PNIPAM had been successfully synthesized by RAFT polymerization using CS-RAFT agent. In addition, it should be noted that the signals of the dodecyl chain of the RAFT agent overlap with the characteristic peaks of PNIPAM. Obviously, two peaks (–CH–CH2) at 1.00–2.20 ppm, a peak (–NH–CH<) at 3.90 ppm and a strong methyl group peak at 1.10 ppm all proved the existence of NIPAM. For the preparation of l-peptide functionalized NPs, the *N*-acetyl groups were removed from the CS-g-PNIPAM by hydrolysis, then EDC and NHS were used to activate the –COOH group of the l-peptide and form an active intermediate which was reacted with the amine group of CS to form a stable amide bond to the l-CS-g-PNIPAM NPs. Successful synthesis of the l-CS-g-PNIPAM NPs was also confirmed by a ^1^H NMR spectroscopy where new signals appear in the ^1^H NMR spectrum of l-CS-g-PNIPAM due to the introduction of l-peptide (Figure 1(C)). The peaks at 8.65 ppm are the typical characteristic signal of aromatics in the l-peptide and indicate that the successful combination of the l-peptide residue with the CS backbone has occurred.

Since the copolymers of l-CS-g-PNIPAM can readily self-assemble and easily encapsulate poorly soluble drugs (Zhang et al., 2016). PTX was successfully loaded into poly l-CS-g-PNIPAM NPs by physical incorporation. They self-assembled in water, in the hydrophobic domains of the NPs, into core-shell nanocarriers at room temperature and neutral pH (7.4), where the water-insoluble CS segments collapse as the cores, and the water-soluble PNIPAM segments form the corona shell. HPLC analysis indicated that DL was 13.5% while EE was 74.3%, suggesting that the relative high EE and DL of l-CS-g-PNIPAM NPs are suitable for delivery of antitumor drugs.

The physicochemical properties of the various kinds of NPs are shown in Table 1. Interestingly, the cores of the PTX-loaded NPs have some influence on particle size, as compared to the blank NPs, PTX-loaded NPs particle sizes are smaller indicating that after the introduction of the drug, the hydrophobic interaction forces between it and the carrier are enhanced resulting in decreased particle sizes. The sizes of all the NPs are below 300 nm and have a narrow size distribution (PDI <0.45), which is appropriate for tumor targeting by the EPR effect, and they have a size distribution that is suitable for intravenous administration (Song et al., 2016; Zhong et al., 2016; Wang et al., 2017).

**Table 1.** Physicochemical properties of CS, *N*-acetyl CS, CS-RAFT, CS-g-PNIPAM, l-CS-g-PNIPAM, and l-CS-g-PNIPAM-PTX, each value represents the mean ± SD (*n* = 3).

	Particle size (nm)	Zeta potential (mV)	PDI	DL (%)	EE (%)
CS	201.5 ± 18.6	31.4 ± 2.8	0.343		
*N*-acetyl CS	226.3 ± 18.3	15.2 ± 1.9	0.417		
CS-RAFT	251.5 ± 21.7	14.1 ± 2.6	0.283		
CS-g-PNIPAM	268.0 ± 20.9	13.9 ± 2.1	0.352		
l-CS-g-PNIPAM	278.2 ± 23.5	19.0 ± 3.2	0.224		
l-CS-g-PNIPAM-PTX	275.4 ± 21.0	18.8 ± 2.0	0.205	13.5 ± 0.9	74.3 ± 5.8

The morphologies of l-CS-*g*-PNIPAM-PTX NPs were investigated by SEM and TEM (Figure 1(D,E)). TEM studies indicate that each nanocarrier has a diffusive boundary outside its solid core, revealing the hydrophilic PNIPAM corona structure, and the results also show the uniformly spherical morphology of l-CS-*g*-PNIPAM-PTX NPs with a diameter which is slightly smaller than that measured by DLS. This may be caused by dehydration during the sample preparation. In addition, it is worth noting that the l-CS-*g*-PNIPAM-PTX NPs are positively charged due to the residual amino groups on CS backbones. It is well known that PNIPAM NPs collapse upon heating showing a negative response to temperature and this property is favorable for the stimuli-responsive release of anti-tumor drugs (Echeverria et al., 2015). In the present study, the evolution with temperature of the measured hydrodynamic diameter (Figure 1(F)) indicates the coincident trend. These results confirm that l-CS-g-PNIPAM-PTX NPs still show thermosensitivity and swelling ability, therefore, the synthesized NPs were suitable for targeted drug delivery.

### MTT analysis

**Figure 2.**
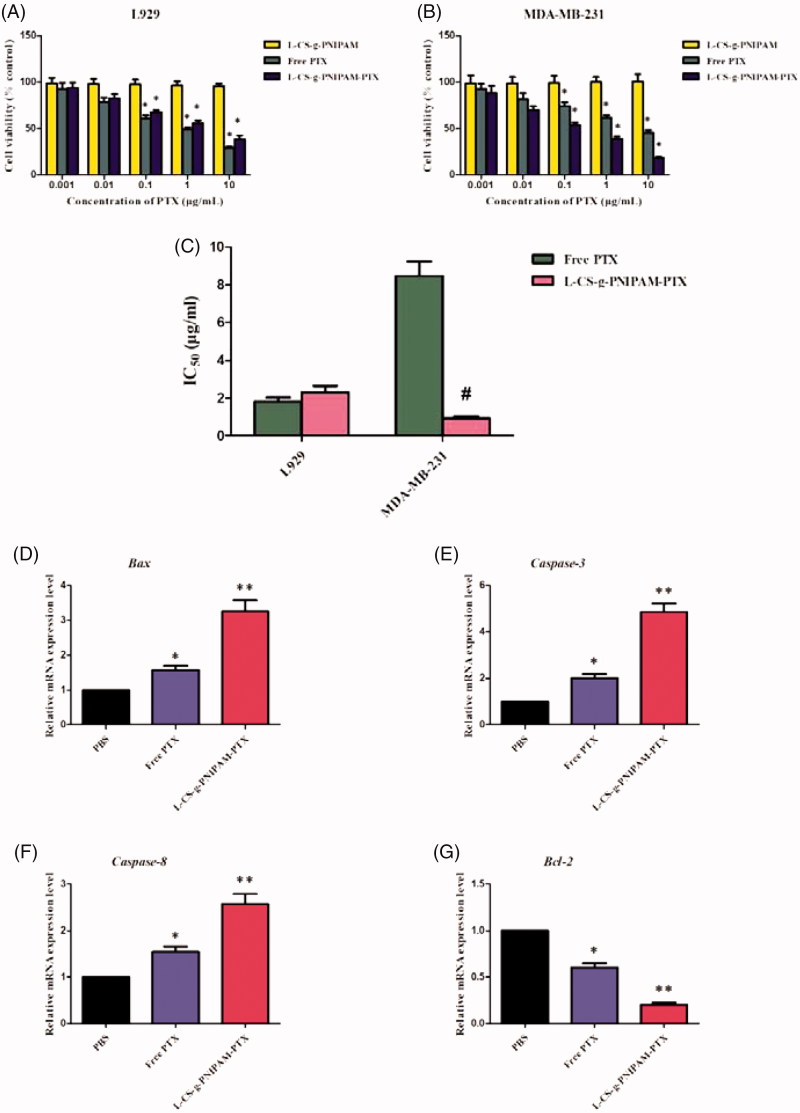
*In vitro* cytotoxic activities of the different formulations against (A) L929 cells; (B) MDA-MB-231 cells; and (C) the IC_50_ of different formulations against L929 cells and MDA-MB-231 cells. Data are expressed as mean ± SD (*n* = 5), *p < .05, compared with l-CS-g-PNIPAM group; **^#^** *p* < .05, compared with free PTX group. The mRNA relative expression levels of (D) *Bcl-2*, (E) *Caspase-3*, (F) *Caspase-8*, and (G) *Bax* in MDA-MB 231 cells. Data are expressed as mean ± SD (*n* = 5), *p < .05, compared with PBS group; **p < .01, compared with PBS group.

An *in vitro* cytotoxicity study, utilizing an MTT assay, was performed to demonstrate the anticancer potential of a series of formulations in MDA-MB-231 breast cancer cell lines and L929 control cell lines. Since PTX has immense potential and is currently one of the drugs of choice for the treatment of breast cancer (Zhang et al., 2016; Song et al., 2017) its effectiveness, using the l-CS-g-PNIPAM NPs system, was explored. As indicated in Figure 2(A,B), MTT tests for blank nanocarriers showed that an increase in the concentration of NPs was not harmful to the survival of both MDA-MB-231 and L929 cells with the cell viability being around 100%. Obvious cytotoxicity of the nanocarriers was not found in the MTT test and noticeable cytotoxicity was not observed after incubation indicating that the biocompatibility of the l-CS-g-PNIPAM was satisfactory. Interestingly, a clear positive correlation was found between cell cytotoxicity (MTT) and concentrations of free PTX and l-CS-g-PNIPAM-PTX. Moreover, there was an obvious reduction in cell survival as the PTX concentration was increased above 0.1 μg/mL in both the MDA-MB-231 and L929 cells. It is worth noting that the cytotoxic effect of l-CS-g-PNIPAM-PTX NPs was greatly enhanced in the MDA-MB-231 cells compared to the L929 cells and the viability of free PTX incubated cells was lower than that of l-CS-g-PNIPAM-PTX incubated cells in L929 cells compared to MDA-MB-231 cells at several concentrations. An important parameter to quantitatively evaluate the *in vitro* anticancer effect of a formulation is to calculate the IC_50_ values (Zhang et al., 2017). This study resulted in an IC_50_ value of 1.83 ± 0.21 and 2.31 ± 0.32 μg/mL in L929 cells for free PTX and l-CS-g-PNIPAM-PTX, respectively, while on the other hand the values were 8.47 ± 0.77 and 0.94 ± 0.10 μg/mL in MDA-MB-231 cells for free PTX and l-CS-g-PNIPAM-PTX, respectively (Figure 2(C)). This significant (*p* < .05) variation in IC_50_ values could be due to the higher uptake of the novel l-CS-g-PNIPAM-PTX NPs by the cancer cell lines. These results all confirmed that the l-CS-g-PNIPAM-PTXNPs were effective in killing these tumor cells and may be promising in future cancer therapy.

### Molecular mechanisms underlying l-CS-g-PNIPAM-PTX NPs treatment-induced apoptosis

In order to investigate the effect of l-CS-g-PNIPAM-PTX NPs on breast carcinoma apoptosis in depth, it was necessary to perform molecular level evaluation. RT-qPCR was used to detect the expression mRNA levels of several pivotal apoptosis-regulatory genes (*Bax*, *Caspase-3*, *Caspase-8*, and *Bcl-2*) in MDA-MB-231 cells. The *Bcl-2* (B-cell lymphoma/leukemia-2) gene is one of the important proto-oncogenes and was the first confirmed gene to be able to participate in anti-apoptosis of cells and prolong cell survival (Yang et al., 1997). The expression of Bcl-2 mRNA in MDA-MB-231 cells after 24 h incubation with PBS, free PTX, and l-CS-g-PNIPAM-PTX was investigated by fluorescence quantitative PCR. As shown in Figure 2(G), compared with the PBS group, free PTX and l-CS-g-PNIPAM-PTX significantly inhibited the expression of *Bcl-2* mRNA in breast cancer cells and promoted the process of cancer cell apoptosis. In addition, the decreased effect of l-CS-g-PNIPAM-PTX was greater than in the free PTX. In contrast, the *caspase-3*, *caspase-8* genes (two members of the cysteine-aspartic acid protease family), and *Bax* (the inhibitor of *Bcl-2*) were activated in the apoptotic cells both by extrinsic and intrinsic pathways, and they were overexpressed in MDA-MB-231 cells after incubation of free PTX or l-CS-g-PNIPAM-PTX. As expected, the l-CS-g-PNIPAM-PTX treated cells exhibited the maximum mRNA content of these three pro-apoptosis genes among all groups (Figure 2(D–G)). The results revealed that the l-peptide functionalized NPs had the potential of inducing apoptosis in tumor cells, however, cell apoptosis is a complex process, regulated by many genes and the apoptotic mechanism of l-CS-g-PNIPAM-PTX at the molecular level needs further in-depth investigation.

### Western blot analysis

**Figure 3.**
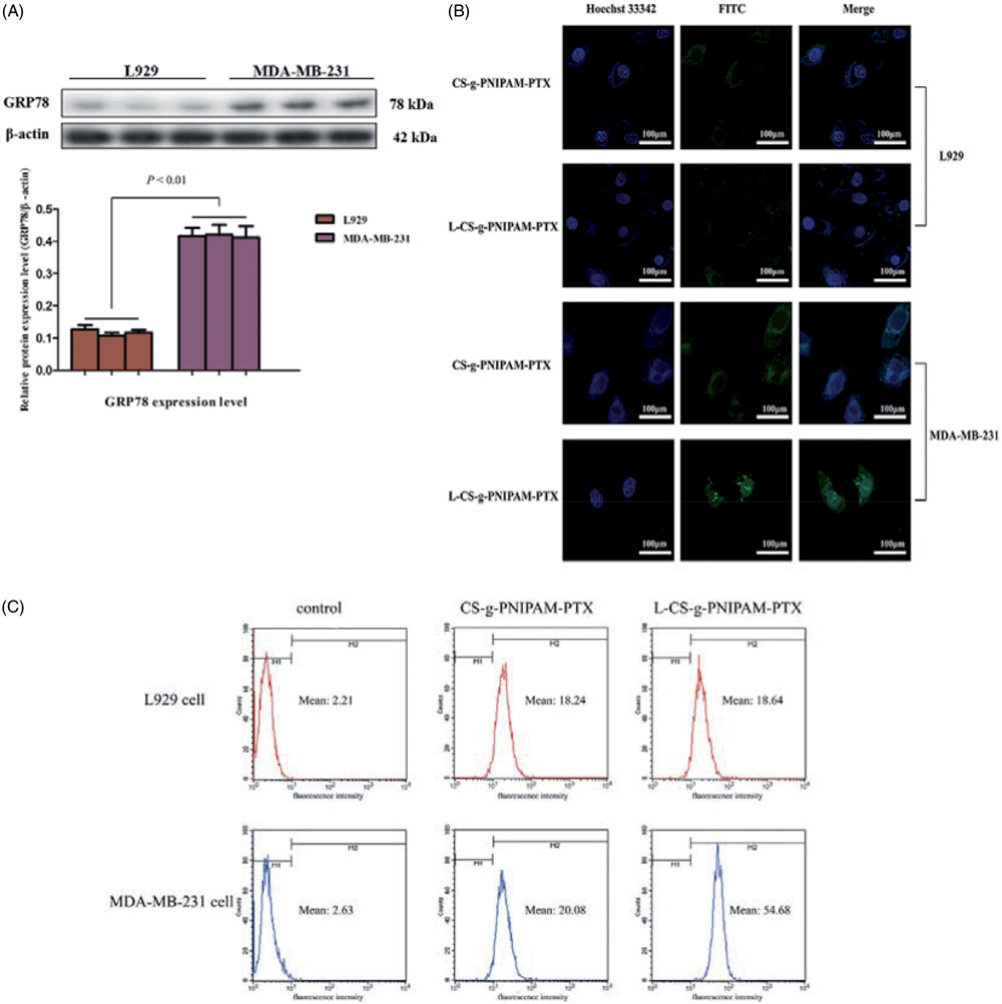
(A) GRP78 protein expression levels in L929 cells and MDA-MB-231 cells. (B) Cellular uptake of CS-g-PNIPAM-PTX and l-CS-g-PNIPAM-PTX NPs measured by confocal in L929 cells or MDA-MB-231 cells; (C) cellular uptake of CS-g-PNIPAM-PTX and l-CS-g-PNIPAM-PTX NPs measured by flow cytometry in L929 cells or MDA-MB-231 cells.

The l-peptide (RLLDTNRPLLPY), discovered through screening of a phage-displayed random peptide library, is reported to bind specifically to GRP78, which has been verified as residing to surface cells of certain cancers. Moreover, the binding capacity of the l-peptide to GRP78 has been finally validated by *in vitro* binding to various cancer cells and *in vivo* tumor imaging and therapeutic studies (Wang et al., 2016). In the present study, l-peptide functionalized dual-responsive NPs were designed and synthesized as a potential chemotherapeutic agent to treat breast tumors. Therefore, the difference in the GRP78 expression levels between malignant breast tumor cells and normal cells required examination and Western blotting assays were conducted to determine the GRP78 protein levels in MDA-MB-231 breast and L929 control cells. As shown in Figure 3(A), GRP78 protein was highly expressed in MDA-MB-231 cells, but with little evidence of expression in L929 cells, indicating that GRP78 could be a binding site for l-CS-g-PNIPAM NPs in breast tumor therapy and that the l-peptide was able to perform its active targeting properties through interaction with GRP78.

### Uptake ability of different kinds of NPs in cells

To assess the cellular uptake and cancer-targeting ability of l-CS-g-PNIPAM-PTX NPs, MDA-MB-231, and L929 cells were cultured with the NPs (with or without l-peptide conjugation), respectively. NPs loaded with PTX was labeled with a fluorescence probe (green) and the nuclei were stained by Hochest 33342 (blue) and the movement and location of the l-CS-g-PNIPAM NPs toward cells was visualized by fluorescence microscopy. Figure 3(B) presents the fluorescence microscope images of cells after incubation with or without l-peptide functionalized CS-g-PNIPAM-PTX NPs and indicates that neither of the agents showed obvious fluorescence in the nucleus of control L929 cells, but fluorescence did appear around the nucleus in the cytoplasm. On the other hand, while CS-g-PNIPAM-PTX NPs showed a weak green fluorescence intensity, the l-peptide functionalized NPs showed a strong fluorescence intensity in the nucleus of MDA-MB-231 breast tumor cells. This indicated that the l-peptide conjugated or not conjugated NPs could enter cells more easily with no special targeting effect in normal cells and they could be readily extruded by the efflux protein on the cell membrane surface, with little adverse impact on cells. As for MDA-MB-231 breast tumor cells, the fluorescence intensity of l-CS-g-PNIPAM-PTX NPs in the nucleus is much stronger compared to CS-g-PNIPAM-PTX NPs, indicating that the GRP78-targeted l-peptide helps the drug to be internalized in cancer cells revealing a virtuous targeting ability. Furthermore, some morphological damage in cells was observed, and the amount of MDA-MB-231 cells is obviously decreased, suggesting that GRP78-targeted l-peptide can intercalate with DNA and is involved in cell apoptosis (Liu et al., 2016). This different intracellular drug uptake behavior can also be attributed to the fast drug release from l-CS-g-PNIPAM NPs by pH-and temperature-triggered shell-shedding in the acid and possibly the hyperpyrexial microenvironment.

To provide further evidence, flow cytometry assay was utilized to determine the cellular uptake properties of the l-CS-g-PNIPAM NPs. CS-g-PNIPAM-PTX and l-CS-g-PNIPAM-PTX NPs were incubated with L929 cells and MDA-MB-231 cells for 4 h and then the cellular uptake of PTX was quantitatively investigated by flow cytometry as shown in Figure 3(C). No significant difference was observed between CS-g-PNIPAM-PTX and l-CS-g-PNIPAM-PTX NPs incubated L929 cell lines, though the fluorescence intensity of the l-CS-g-PNIPAM-PTX NPs treated group (mean fluorescence intensity: 54.68) was significantly increased compared to the CS-g-PNIPAM-PTX NPs treated group (mean fluorescence intensity: 20.08) in MDA-MB-231 cells, which is consistent with the results of confocal microscopic analysis. All these cellular uptake results demonstrate that l-CS-g-PNIPAM NPs are able to deliver more PTX into tumor cells due to the active targeted effect of the l-peptide.

### Controlled PTX release

**Figure 4.**
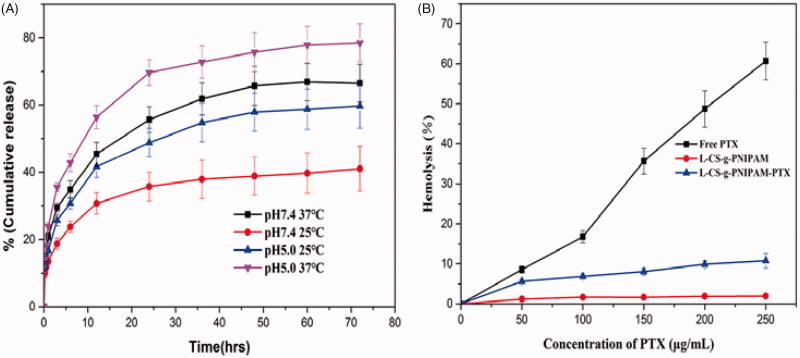
(A) *In vitro* PTX release in PBS at different pH and different temperatures, as measured by a dialysis method. The increase of PTX concentration was determined by HPLC (*n* = 3). (B) Hemolytic activity of free PTX, l-CS-g-PNIPAM and l-CS-g-PNIPAM-PTX NPs on mice red blood cells (*n* = 3, mean ± SD).

The effect of environmental stimuli on the release of the drug from l-CS-g-PNIPAM-PTX was studied by dialysis, by incubating the self-assembled nanocarriers in buffers at different temperatures (37 °C, 25 °C) and pH (5.0, 7.4). Figure 4(A) shows the cumulative release of PTX over a period of three days at pH =7.4 and pH 5.0, from the l-CS-g-PNIPAMNPs and it can be seen that the drug release was slow at pH 7.4 while it was accelerated at lower pH. Interestingly, low release of PTX is observed under conditions mimicking storage and transit over a 72 h period at neutral pH (7.4) when the PTX is encapsulated inside an insoluble CS core. By contrast, PTX is released at a remarkably enhanced rate under acidic conditions mimicking a tumor pH (5.0) due to the fact that the core-shell nanocarriers are reversed under these conditions and the CS based copolymers are exposed to acid degradation. After the initial rapid release phase, the profile gradually plateaus with prolonged PTX release at a much slower rate. This is because of acid digestion of the CS-g-PNIPAM conjugated chain segments that are buried inside the PNIPAM cores during the transition, which gradually become exposed to the surface of the PNIPAM cores due to its immiscibility with PNIPAM and constant polymer chain movement. Figure 4(A) also shows the significant change in release of PTX from l-CS-g-PNIPAM NPs at different temperatures. Within 72 hours, only about 62% PTX was released from l-CS-g-PNIPAM NPs at 25 °C, but at 37 °C, release of PTX was enhanced to 83%. The increased early stage release rate at both 25 °C and 37 °C is due to the availability of unbound drug on the surface of the l-CS-g-PNIPAM NPs. However, during the later stage, PTX release was reduced at 25 °C due to a stable hydrophilic segment of the PNIPAM units on the nanocarriers. However, when the temperature is increased to 37 °C, the PTX release rate is increased due to the collapse of the structure of copolymers possibly due to temperature-induced structural modification of the l-CS-g-PNIPAM NPs triggered by the phase transition of the PNIPAM. Based on the release profile data, these pH- and temperature-responsive drug carriers appear to be most suitable for cancer treatment.

### Blood compatibility

Although PTX is a very effective anticancer drug there still exists the challenge of inherent toxicity and one of the major aims of the current work was to address and attempt to reduce the toxicity posed by PTX through use of a biodegradable polymer such as l-CS-g-PNIPAM. The pharmaceutical application of the l-CS-g-PNIPAM-PTX NPs *in vivo* depends on several characteristics, including safety, stability, and blood compatibility since they are usually administered via intravenous injection. The hemocompatibility of the NPs was therefore investigated and the hemolytic curves for free PTX, l-CS-g-PNIPAM NPs and PTX loaded l-CS-g-PNIPAM NPs at different concentrations of PTX are shown in Figure 4(B) (during the experiments, the concentrations of PTX in the two formulations were kept the same and therefore the concentrations of l-CS-g-PNIPAM and l-CS-g-PNIPAM-PTX NPs are different). The results suggest that free PTX causes extensive damage to RBCs, while l-CS-g-PNIPAM and l-CS-g-PNIPAM-PTX NPs showed dramatically lower levels of hemolysis, illustrating the excellent blood compatibility of l-CS-g-PNIPAM NPs as well as their potential application as drug delivery carriers.

### *In vivo* anti-tumor effects

**Figure 5.**
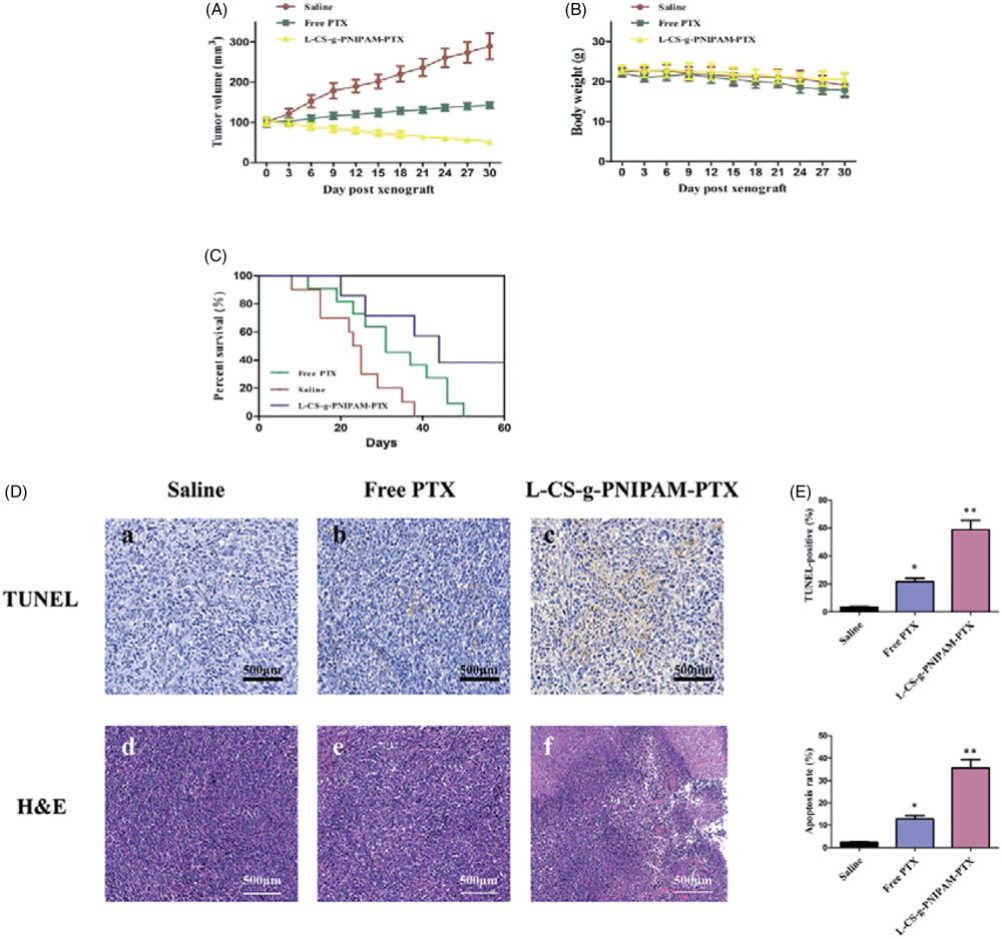
*In vivo* tumor therapy characterization: (A) tumor growth after systemic administration of different drug treatments to mice bearing MDA-MB-231 (mean ± SD, *n* = 10); (B) *in vivo* toxicity (in terms of body weight lost) of saline (control), free PTX, and l-CS-g-PNIPAM-PTX on mice bearing MDA-MB-231 tumors after a schedule of multiple doses; (C) Kaplan–Meier’s survival curve of tumor-bearing mice after different treatments. (D) Representative photomicrographs of tumors with TUNEL (a–c), as well as H&E staining of tumor sections (d–f); (E) quantitative analysis of TUNEL-positive and cell apoptosis in each group (mean ± SD, *n* = 10), *p < .05, compared with saline group; **p < .01, compared with saline group.

Finally, the therapeutic performance of l-CS-g-PNIPAM-PTX NPs using MDA-MB-231 human breast tumor-bearing nude mice was evaluated. The mice were treated with l-CS-g-PNIPAM-PTX or PTX (10 mg PTX equiv/kg) every three days after the tumors grew to about 100 mm^3^ in volume and physiological saline were used in the control group. Thirty days post-injection, the tumor size of mice treated with saline was substantially larger with an average volume of 289.9 ± 32.9 mm^3^ and average bodyweight of 19.1 ± 2.0 g (Figure 5(A,B)). PTX treatment succeed in postponing the growth of tumor tissue and the tumor body average volume and weight were 142.7 ± 7.6 mm^3^ and 17.9 ± 1.4 g, respectively, which is about half of that for the saline-treated mice. Strikingly, the average tumor size of l-CS-g-PNIPAM-PTX-treated mice was statistically significant and much lower than that of PTX-treated mice with an average tumor volume and body weight were 51.7 ± 5.0 mm^3^ and 20.5 ± 1.7 g, respectively and significantly, the treatment had little adverse effect on the mice physiology. Consistently, the treatment with l-CS-g-PNIPAM-PTX prolonged the survival period of MDA-MB-231 tumor-bearing mice to a much greater extent than that in the other groups (Figure 5(C)). Thus, l-CS-g-PNIPAM-PTX NPs exhibited excellent targeting ability and effective treatment of MDA-MB-231 human mammary carcinoma *in vivo*, showed high treatment efficacy and significantly reduced systemic toxicity.

### Histological analyses of tumor apoptosis

The histological analyses of tumor growth inhibition by this NPs, TUNEL apoptosis assay, and H&E staining were performed. TUNEL assays revealed that l-CS-g-PNIPAM-PTX NPs induced wide spread apoptosis of tumor cells, on the other hand, nearly no cell apoptosis (claybank dots) was observed in groups treated with saline, and few of TUNEL positive sites were found in groups treated with free PTX, suggesting that this system could inhibit tumor growth through inducing cells apoptosis in solid tumor (Figure 5(D)(a–c)). The similar tendency was also observed in H&E staining assay (Figure 5(D)(d–f)). The results of H&E staining showed that l-CS-g-PNIPAM-PTX NPs caused severe necrosis in the tumor tissue. On the contrary, less tumor cell death was observed for mice treated with saline or free PTX. Figure 5(E) shows the quantitative analysis of the TUNEL-positive and cell apoptosis in each group, and it can be seen that l-CS-g-PNIPAM-PTX NPs induced significant cell apoptosis in the tumor tissue. These results confirm that l-CS-g-PNIPAM-PTX NPs could effectively target to GRP78 positive human breast tumor xenografts, kill cancer cells by both apoptosis and necrosis mechanisms.

## Conclusions

In summary, a thermally responsive PNIPAM has been grafted onto the CS via a facile RAFT process and the CS-g-PNIPAM copolymer conjugate self-assembles into nano-spheres with a favorable size distribution and smart stimuli-responsive drug release profiles that are highly desired for intravenous administration. Moreover, l-peptide coated with PTX loaded CS-g-PNIPAM NPs can induce potent antitumor effects *via* both intra-tumor and systemic administration. Furthermore, l-CS-g-PNIPAM-PTX NPs achieved greater cancer cell killing efficacy than free PTX, establishing that this formulation can further enhance the synergistic therapeutic effect of a combination treatment. These results were reproduced *in vivo*, where l-CS-g-PNIPAM-PTX NPs induced an antitumor effect by enhancing the accumulation of oncolytic drug and induction of apoptosis in tumor tissue. Taken together, the use of l-CS-g-PNIPAM-PTX NPs is a promising strategy for inducing systemic antitumor effects in future breast cancer clinical trials.
